# Multimodality Imaging of Calcinosis of Chronic Renal Failure

**DOI:** 10.7759/cureus.1113

**Published:** 2017-03-24

**Authors:** Raul Loya, Kimberly Beavers, Kurt Scherer

**Affiliations:** 1 Diagnostic Radiology, Florida Hospital-Orlando

**Keywords:** tumoral calcinosis, imaging, chronic renal failure, ultrasound, secondary tumoral calcinosis

## Abstract

Secondary tumoral calcinosis (STC) refers to periarticular calcified masses associated with an identifiable condition. The most common of these identifiable conditions is a chronic renal failure. We present a unique case in which massive periarticular masses in a patient with calcinosis of chronic renal failure (CCRF) are demonstrated in the shoulder and hip on sonography, radiography and computed tomography (CT).

## Introduction

The prevalence of periarticular masses in end-stage-renal-disease (ESRD) patients is 0.5%-1.2% [1]. Calcinosis of chronic renal failure (CCRF), also referred as secondary tumoral calcinosis (STC), is indistinguishable radiologically and histologically from primary tumoral calcinosis (PTC). Laboratory values and history of ESRD are distinguishing factors in making the diagnosis [1]. PTC patients have normal serum calcium levels and elevated phosphate levels. CCRF patients have decreased calcium levels and increased phosphate levels.

## Case presentation

A 45-year-old male with ESRD and secondary hyperparathyroidism initially presented with hip and shoulder pain. Multimodality imaging including radiography, sonography, and computed tomography (CT) illustrated findings typical of CCRF. STC patients have decreased calcium and increased phosphorus levels, as with our patient who presented with slightly decreased calcium levels (8.1 mg/dl) and elevated phosphorus (5.7 mg/dl).

**Figure 1:**
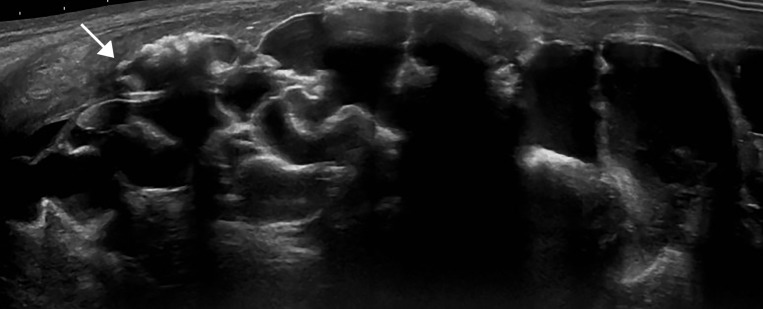
Right hip ultrasound Grayscale ultrasound image demonstrating large, echogenic, lobulated calcifications (arrow) in the lateral right hip.

**Figure 2:**
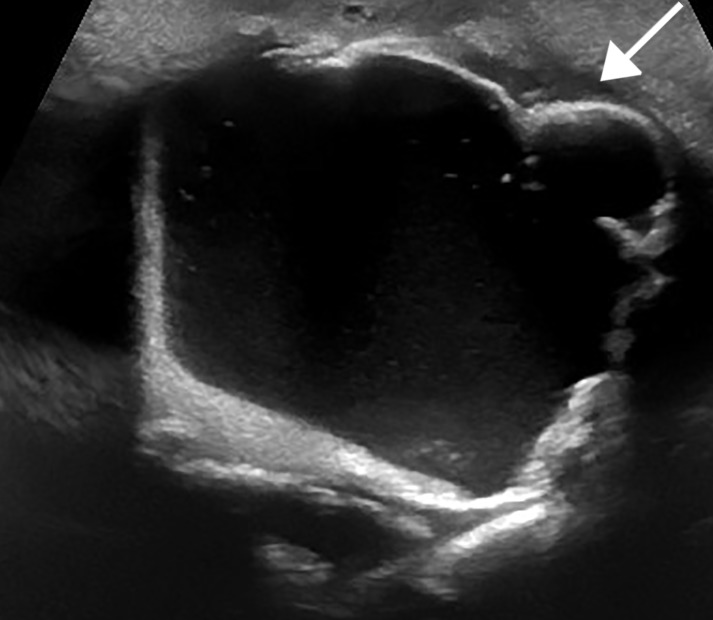
Right shoulder ultrasound Grayscale ultrasound image demonstrating large, echogenic, lobulated calcifications (arrow) in the lateral right shoulder.

**Figure 3:**
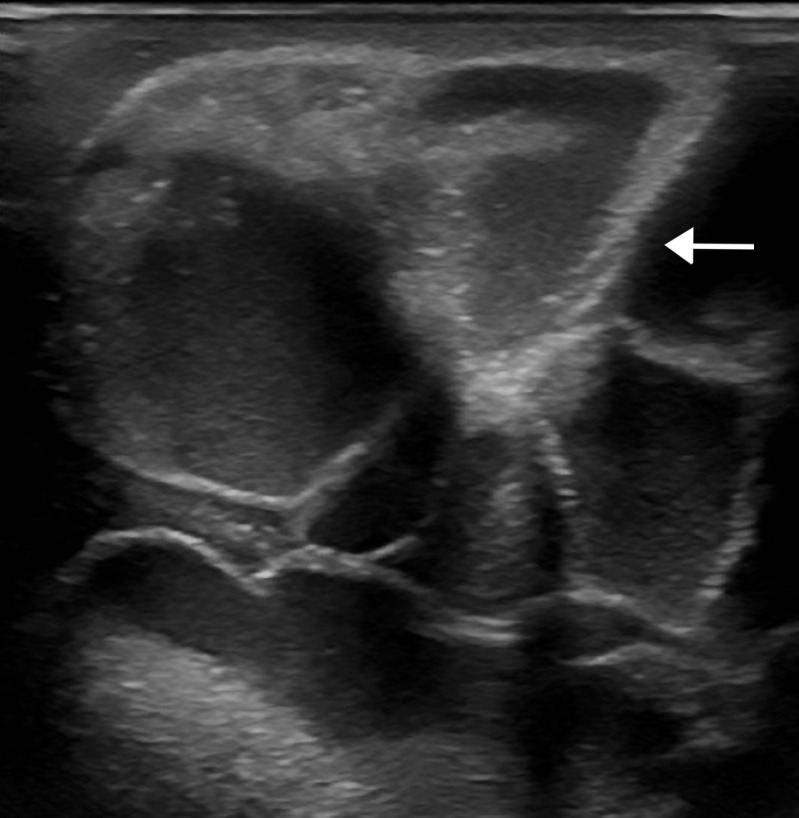
Right forearm ultrasound Grayscale ultrasound image demonstrating large, echogenic, lobulated calcifications (arrow) in the dorsal proximal right forearm.

Ultrasound findings of CCRF are echogenic, large, lobulated calcifications. Massive periarticular masses are demonstrated on ultrasound involving the right hip (Figure 1) and right shoulder (Figure 2). Additional manifestations are also present in the right forearm (Figure 3).

**Figure 4:**
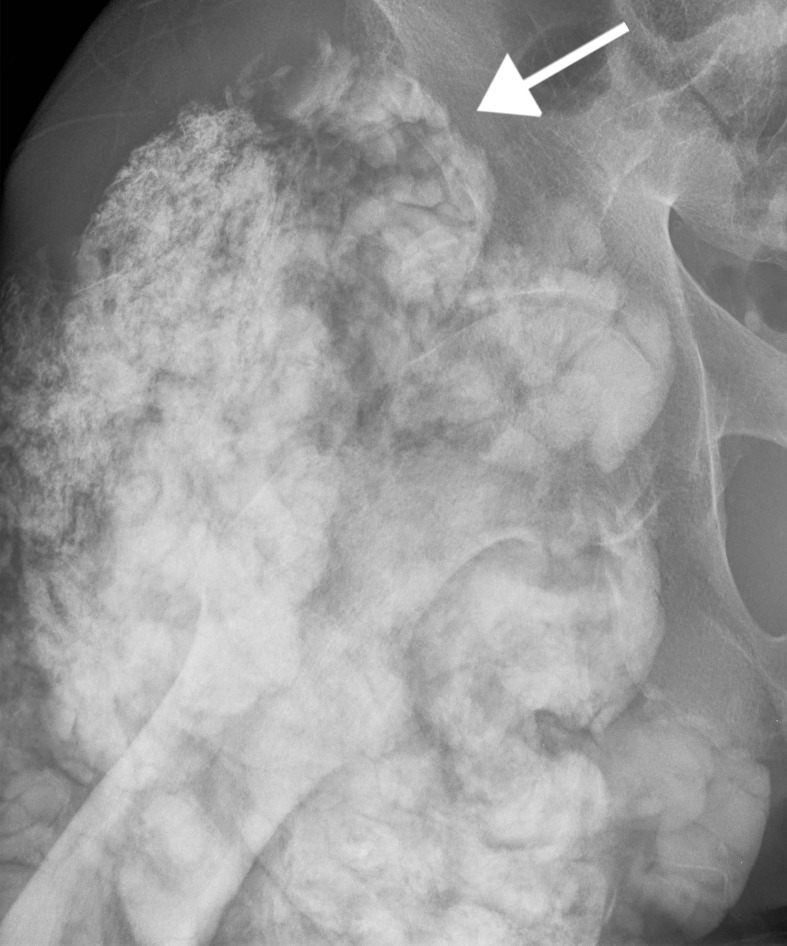
Right hip radiograph Anteroposterior (AP) radiograph of the right hip demonstrating large, dense, lobulated calcifications in a periarticular distribution (arrow).

**Figure 5:**
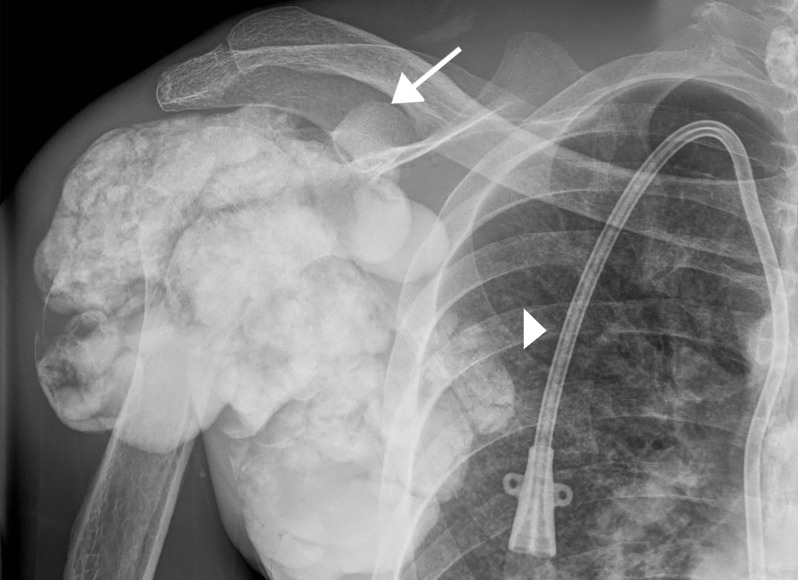
Right shoulder radiograph AP radiograph of the right shoulder demonstrating large, dense, lobulated calcifications in a periarticular distribution (arrow). Double lumen dialysis catheter (arrowhead).

**Figure 6:**
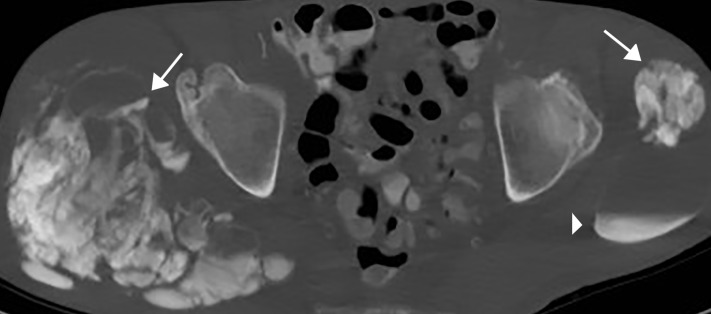
Bilateral hip CT Axial CT image at the level of the hips demonstrates bilateral, dense, multilobulated periarticular masses (arrows). Sedimentation sign (arrowhead).

**Figure 7:**
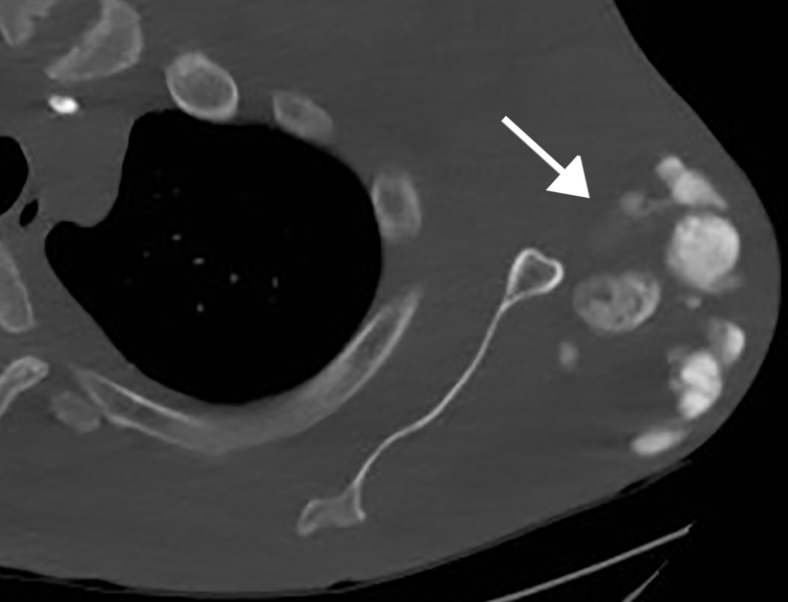
Left shoulder CT Axial CT image at the level of the left shoulder demonstrates dense multilobulated periarticular masses (arrow). Note lack of osseus destruction in the adjacent left scapula.

Radiographic findings are amorphous, cystic and lobulated (cloud-like), dense calcifications in a periarticular distribution [2]. This is demonstrated in our patient at the right hip (Figure 4) and right shoulder (Figure 5). Some of the cystic masses may show fluid-fluid levels caused by calcium layering, also known as the sedimentation sign [3]. This may be seen on all modalities and is demonstrated in our patient on CT (Figure 6). CT can better delineate the masses and will typically demonstrate lack of erosion or osseous destruction, which is a classic feature of tumoral calcinosis (Figure 7). This is an important characteristic that helps differentiate STC from neoplastic entities such as osteosarcoma, chondrosarcoma and synovial sarcoma [4].

Two patterns are generally observed with T2 sequences on magnetic resonance imaging (MRI). Either a diffuse, low signal intensity pattern or a nodular pattern with alternating areas of high signal intensity and signal void [1]. T1-weighted sequences usually show heterogeneous lesions with low signal intensity [1]. Magnetic resonance images were not obtained for our patient.

## Discussion

Patients with tumoral calcinosis (TC) usually present with periarticular masses associated with pain and joint movement limitation commonly affecting the hip, elbow, and shoulder [2, 4]. It most commonly involves the greater trochanteric bursa [1]. Diagnosis is difficult with imaging alone and it must rely on a combination of typical radiologic features and biochemical profile. It is also important to exclude connective tissue diseases such as scleroderma and dermatomyositis, which may show periarticular calcifications similar to TC [4].

As discussed, a distinguishing imaging feature of CCRF is the sedimentation sign. An advantage of using ultrasound is that layering calcium may be identified on this modality when it is not seen on the radiograph or CT [5]. This is helpful in diagnosing CCRF. Additionally, ultrasound offers a modality in which the patient is not exposed to radiation to confirm the presence of CCRF. This may be especially useful in cases similar to ours in which there are multiple periarticular masses in a patient with ESRD. Once CCRF has been confirmed in one joint, ultrasound may be used to confirm STC in the other joints. Finally, ultrasound is an inexpensive modality when compared to radiograph, CT or MRI.

Medical treatment is preferred over surgery in patients with STC. Surgical interventions should be kept as a last resort in these patients [2]. Surgical excision is associated with a high recurrence rate and postsurgical complications that include infection and fistula formation [2].

Multifactorial calcification of TC is initiated by elevated calcium-phosphorus product with hyperphosphatemia as the main component [2]. Medical treatment includes calcium and phosphorus restricted diets that decrease the calcium-phosphorus product. Other options are dialysates and phosphate binders [2].

Other medications such as sodium thiosulfate and intravenous bisphosphonates have been used in the treatment of STC with variable success rates [2]. Given the underlying secondary hyperparathyroidism in these patients, parathyroidectomy may also be considered in the setting of medical treatment failure. This approach has demonstrated significant response [2]. Kidney transplantation is another consideration [2].

## Conclusions

Our case offers a unique perspective as ultrasound findings are not widely described in the literature. Additionally, as seen in this patient, ultrasound offers an inexpensive clinically useful modality for identifying the etiology of soft tissue masses in ESRD patients, without the risks of radiation exposure.
